# A randomized, double blind, parallel, placebo‐controlled study to investigate the efficacy of *Lactobacillus paracasei* N1115 in gut development of young children

**DOI:** 10.1002/fsn3.2533

**Published:** 2021-09-01

**Authors:** Shijie Wang, Yiping Xun, Grace J. Ahern, Lili Feng, Dong Zhang, Yuling Xue, Reynolds Paul Ross, Andrea M. Doolan, Catherine Stanton, Hong Zhu

**Affiliations:** ^1^ Shijiazhuang Junlebao Dairy Co. Ltd. Shijiazhuang China; ^2^ Hebei University of Science & Technology Shijiazhuang China; ^3^ School of Microbiology University College Cork Cork Ireland; ^4^ APC Microbiome Institute University College Cork Cork Ireland; ^5^ Atlantia Food Clinical Trials Ltd. Cork Ireland; ^6^ Teagasc Moorepark Food Research Centre Fermoy Ireland

**Keywords:** caesarean section, fecal microbiota, *Lactobacillus paracasei* N1115, probiotic, short‐chain fatty acid

## Abstract

In this clinical trial, the safety and effectiveness of *Lactobacillus paracasei* N1115 (LP N1115) were investigated as a potential probiotic to enhance gut development in young children born by caesarean section. Infants and young children between the ages of 6 months and 3 years were administered with a probiotic consisting of LP N1115 strain (*n* = 30) or placebo supplement (*n* = 30) over an 8 weeks intervention. And the stool consistency, bowel habits, salivary cortisol, fecal microbiota, and short‐chain fatty acid metabolism were investigated. Efficacy data were obtained from 58 participants who completed the study. Overall, the placebo functioned similarly to LP N1115 group in relation to stool consistency, gastrointestinal symptoms, salivary cortisol, and short‐chain fatty acids. However, the scoring data relating to the 6–18 months subgroup receiving LP N1115 remained stable over 8 weeks in comparison to placebo. Analysis of the fecal microbiota using 16S rRNA amplicon sequencing revealed that the phyla Firmicutes represented 62% of the microbial relative abundance in the feces of the subjects during the intervening period. No significant changes in alpha‐ or beta‐diversity were noted between the placebo and LP N1115 groups overtime and at each time point. Differential abundance analysis indicated an increase in *Lactobacillus* in LP N1115 group at weeks 4 (*p* < .05) and 8 (*p* < .05) in comparison to the placebo group. These results suggest that probiotic supplementation with LP N1115 was well tolerated by the young children and subtle changes in the microbiome were noted throughout the intervention period.

## Graphical abstract

A randomized, double‐blind, parallel, placebo‐controlled study on *Lactobacillus paracasei* N1115 (LP N1115) was investigated as a probiotic to enhance gut development in young children born by caesarean section. Infants and young children between the ages of 6 months and 3 years were administered with a probiotic consisting of LP N1115 strain (*n* = 30) or placebo supplement (*n* = 30) over an 8 weeks intervention. The study results would seem to indicate that efficacy of LP N1115 supplementation at an early age may be effective in enhancing the gut development.

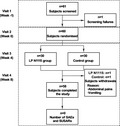

## 1 INTRODUCTION

Delivery by caesarean section (C‐section) is a common obstetrician procedure, and over the past two decades, C‐section delivery by maternal request has increased dramatically. However, a number of physiological risk factors are often associated with this mode of delivery such as postpartum sepsis, hemorrhage, and some morbidities relating to the neonate, and predisposition to illness later in childhood may include respiratory distress, delayed maternal adaptation, obesity, and allergy (Betrán et al., 2016; Gupta & Saini, 2018). In recent years, researches have shown that the prevalence of C‐section also coincides with an aberrant gut microbiota development in the neonate.

There is an ever‐increasing realization that bacterial communities present in the gut during early life play crucial roles in gastrointestinal maturation, immune development, and metabolism (Arrieta et al., 2014; Clemente et al., 2012; Rodríguez et al., 2015). Early microbial colonization of bacteria, archaea, eukaryotes, bacteriophages, and viruses is considered a nonrandom essential process that is influenced by a series of prenatal and postnatal factors including mode of delivery (Dominguez‐Bello et al., 2010; Hill et al., 2017; Neu & Rushing, 2011), gestational age at birth (Fouhy et al., 2019; Hill et al., 2017), feeding type (Bezirtzoglou et al., 2011), maternal health status (Collado et al., 2010), and exposure to intrapartum and postnatal antibiotics (Azad et al., 2016; Tapiainen et al., 2019). The microbial composition of spontaneous vaginally delivered infants differs significantly from C‐section delivered infants (Azad et al., 2014; Bäckhed et al., 2015; Hill et al., 2017) and this is also the case for associated functional metabolites (Hill et al., 2017). Increased relative abundances of Bacteroides and Lactobacillus species are indicative of infants born vaginally as they are directly exposed to the mother's vaginal microbiota. In the case of C‐section delivery, specifically elective caesarean, vertical microbial transmission between the mother and infant is less pronounced; thus, these pioneer bacterial species are often depleted or delayed in the gut of these infants. Inevitably, bacteria are acquired from other body regions of the mother including the skin and mouth or nonmaternal sources such as the hospital environment (Bäckhed et al., 2015; Milani et al., 2017; Shin et al., 2015). It is known that abnormal colonization patterns and concomitant antibiotic administration during infancy favor the growth of opportunistic pathogens and subsequent infection (Shao et al., 2019). Thus, alterations to the gut microbiota may have long‐term implications with increased susceptibility to noncommunicable diseases but not in all cases. These include allergic disease (Bager et al., 2008), immune‐related disorders (Sevelsted et al., 2015), chronic inflammatory disease (Bager et al., 2012; Mårild et al., 2012), obesity (Kuhle et al., 2015), and type 1 diabetes (Cardwell et al., 2008).

The clinical relevance of the changes that occur as a result of C‐section and the impact on later microbiota–immune homeostasis is still unclear and therapeutic interventions are limited. One effective strategy for positively modulating stress, microbial balance, and gastrointestinal symptoms is through the use of single‐ and multistrain probiotics with proven health benefits. Lactobacillus and Bifidobacterium strains/species are recognized as safe, natural components of the gut microbiota and fermented dairy formulations. Encouraging evidence illustrates that dietary supplementation utilizing these commensal constituents or, indeed, their protective metabolites are effective in partially attenuating disease progression and, in turn, stimulate the growth of health‐promoting bacteria in the colon of adults and infants (Di Cerbo et al., 2016; Mathur et al., 2020). Despite the lack of knowledge surrounding the mechanisms of action, it is generally accepted that probiotics function in a strain‐specific manner. Thus, attempts to supplement young, vulnerable pediatric populations are considered desirable and, in doing so, it could provoke distinctive modulatory effects which may promote biological resilience against intestinal infection and acquisition of more favorable microorganisms throughout the life span.

In this randomized, double‐blind, placebo‐controlled study, our aim was to investigate the efficacy and safety of *Lactobacillus paracasei* (LP N1115) as a probiotic to enhance digestive symptoms, salivary cortisol levels, gut microbiota composition, and short‐chain fatty acids levels in infants and young children born by C‐section. In a previous study, it was found that consumption of a yogurt supplemented with LP N1115 for 12 weeks could reduce the risk of acute upper respiratory tract infections in healthy people (aged ≥45 years), via enhancing T cells (${\text{CD}}_{3}^{+}$) that mediate the natural immune defense system (Guo et al., 2016; Pu et al., 2017). Subsequent studies have demonstrated its effectiveness in enhancing intestinal development in neonatal mice and its ability to increase defecation volume, moisture content, and intestinal propulsion rates in a constipated mouse model (Cao et al., 2018; Wang et al., 2016).

## 2 MATERIALS AND METHODS

### 2.1 Probiotic sample

The LP N1115 probiotics contained a single, live strain of *L. paracasei* N1115 (probiotic sample, 2.4 × 10^9^ CFU/g) and the reference product contained maltodextrin without a bacterial strain (placebo sample) which were all provided by Shijiazhuang Junlebao Dairy Co. Ltd. All samples were stored at 4℃, as well as away from direct heat and sunlight.

### 2.2 Subjects

The healthy infants/young children were selected according to strict criteria, and all subjects were males and females aged ≥6 months and ≤3 years, who were born by C‐section. Exclusion criteria included: (i) consumption of probiotics and/or prebiotics in any form within the past 2 weeks, (ii) unwillingness to avoid other probiotics/prebiotics for the duration of the study, (iii) food allergies or an allergy or hypersensitivity to any component of the study products including milk protein allergy or cow's milk allergy, (iv) significant acute or chronic coexisting illness (cardiovascular, gastrointestinal, endocrinological, immunological, metabolic, or any condition which contraindicates, in the investigators judgment, entry to the study), (v) usage of antibiotics within the past 3 months prior to participation in the study, (vi) enrolment in another clinical trial not less than 60 days prior to this study, and (vii) exposure to any nonregistered drug product within 30 days prior to this study. Fulfilment of inclusion and exclusion criteria was validated by the investigator and appointed research nurse.

### 2.3 Study design

**FIGURE 1:**
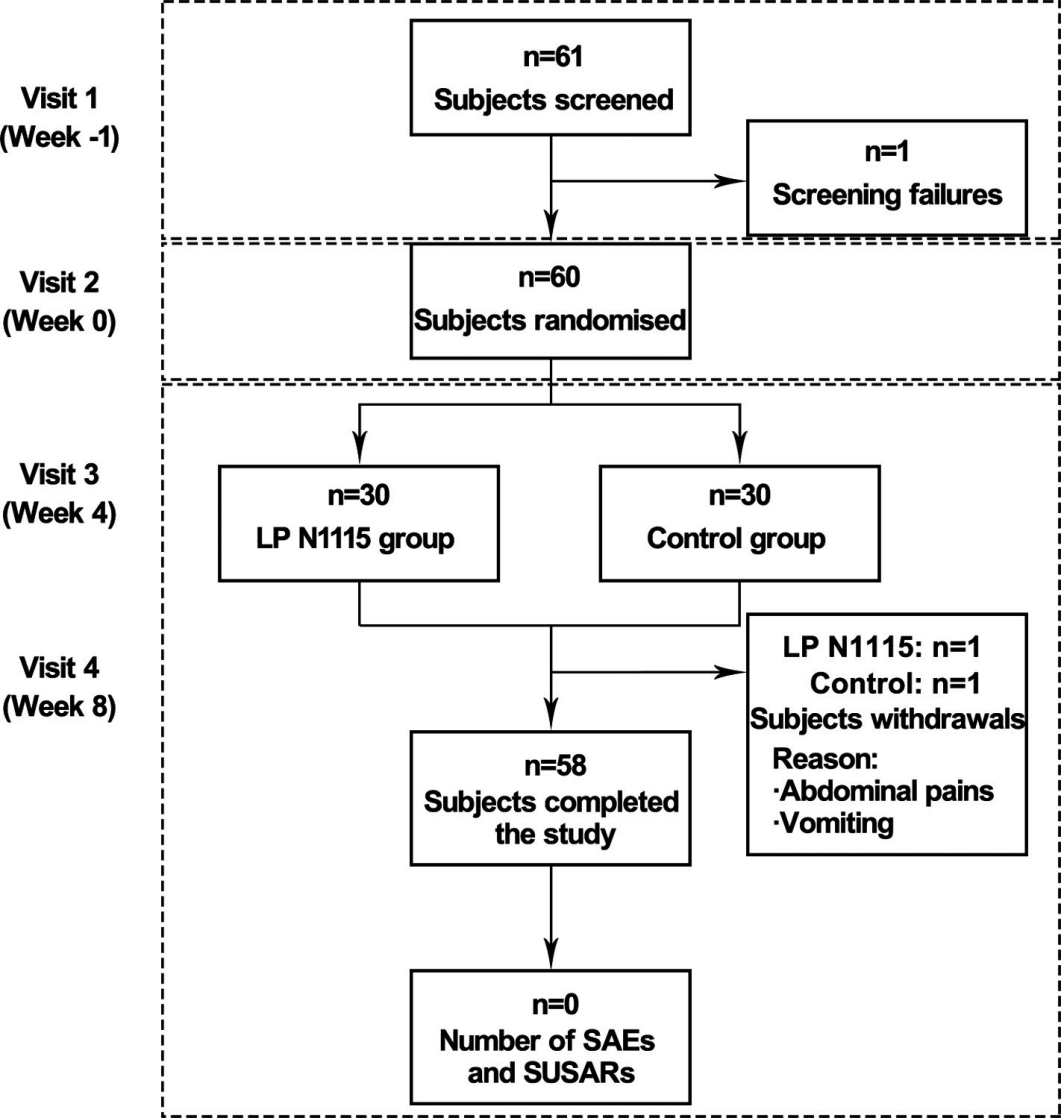
A flow chart showing disposition of subjects. The study involved four visits over an 8‐week period. Values are expressed as the number of subjects. SAEs, Serious adverse events; SUSARs, Suspected Unexpected serious adverse reactions

The study followed the design of a randomized, double‐blind, parallel, placebo‐controlled clinical trial, which involved at least four visits over an 8‐week period (Figure 1). Healthy subjects were randomized to two groups and given the probiotic or placebo samples once a day (2 g/day dose) with warm water or milk. The parent/guardian needed to record the number of bowel movements the subjects had each day, the stool consistency, and if they experienced any abdominal pain, bloating, or flatulence (gas). Stool and saliva samples were also collected at the study site during three visits (visits 2, 3, and 4) for analysis of microbiota composition, short‐chain fatty acids (SCFAs), and cortisol.

The study was approved by the Clinical Research Ethics Committee of the Cork Teaching Hospitals (CREC, Ireland), and written, informed consent was obtained from all parents/guardians on behalf of the child. The trial was conducted in accordance with the ethical principles set forth in the current version of the Declaration of Helsinki (seventh revision; October 2013). The study was registered at ClinicalTrials.gov (identifier: NCT03416595).

### 2.4 Salivary cortisol analysis

A saliva sample was collected by a research nurse using a cotton swab from the subject's mouth. Samples were centrifuged and stored at −80℃ until used. Salivary cortisol was measured using a commercial enzyme‐linked immunosorbent assay kit (Enzo Life Sciences).

### 2.5 Fecal microbiota analysis

Fresh fecal samples were collected by parent/guardian for microbiota and metabolomic analysis. Microbial DNA was extracted using the repeat bead beating plus column method described by Yu and Morrison (2004) with some modifications. Libraries were prepared with Illumina 16S metagenomic sequencing library preparation guide and sequenced on the MiSeq sequencing platform adhering to standardized Illumina protocols (Edgar, 2010). OTUs were aligned using PyNAST and taxonomy was assigned using BLAST against the SILVA SSURef database release v123.

### 2.6 SCFAs analysis

Fecal sample was diluted in 2× wt/vol of sterile Dulbecco's Phosphate buffer saline. The filter was removed before storing the fecal water at −20℃ until gas chromatography mass spectrometry (GC‐MS). Fecal waters were sent to MS‐OMICS for targeted SCFAs analysis containing 10 different compounds (acetic acid, formic acid, propanoic acid, 2‐methylpropanoic acid, butanoic acid, 3‐methylbutanoic acid, pentanoic acid, 4‐methylpentatoic acid, hexanoic acid, and heptanoic acid).

### 2.7 Statistical analysis

The study was analyzed according to the intention to treat, which comprised all randomized subjects who have taken at least one dose of treatment. In case of missing data, excepting those related to the daily diary, last observation carried forward method was applied to conduct the statistical analysis. Descriptive analyses and mixed analysis of variance models (ANOVAs) with Tukey post hoc analyses were conducted as appropriate for all primary and secondary efficacy variables including flatulence, bloating, abdominal pain, SCFAs, and salivary cortisol. Where assumptions of mixed ANOVAs were breached, a mixed ANOVA using 20% trimmed means was performed. Fecal microbiota composition was analyzed using the web application MicrobiomeAnalyst (available at: http://www.microbiomeanalyst.ca) (Dhariwal et al., 2017), and raw OTU sequence counts were subjected to cumulative sum scaling normalization to account for the differences in sequencing depth across the samples. In addition, taxa with less than 0.01% relative abundance across all samples were excluded. All statistical analyses were calculated using SPSS v.24 and R Studio v.1.1.456. A *p*‐value of ≤.05 was considered a significant difference.

## 3 RESULTS

### 3.1 Demographics and intestinal movements

**FIGURE 2:**
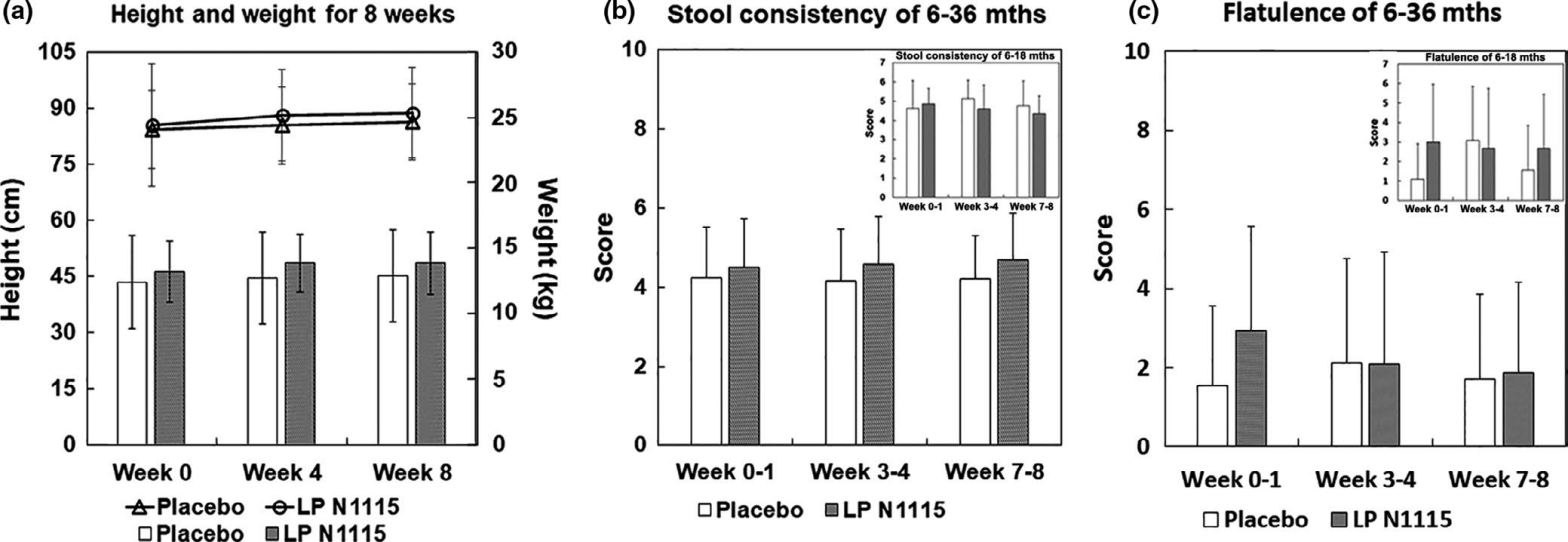
The anthropometrics and intestinal movements of subjects. (a) The height (line chart used the left vertical axis) and weight (histogram used the right axis) of subjects in both groups showed no significant differences. (b, c) The scores of stool consistency and flatulence were calculated based on daily symptom diary. Both of them revealed no significant differences, so were the results in the 6–18 months subgroup shown in the upper right corner of each histogram

Between October 2017 and March 2018, a total of 61 subjects were screened for eligibility, of which one subject was lost to follow‐up and, therefore, not randomized. The remaining 60 subjects were randomly assigned to the LP N1115 group (*n* = 30) and the control group (*n* = 30), respectively (Figure 1). During the study, two subjects withdrew before completion of the trial due to vomiting and abdominal pain. Randomization was well balanced and the anthropometrics were presented (Figure 2a). Besides, two subgroups of age were divided to 6–18 months subgroup (*n* = 22, 10 subjects in LPN1115 group and 12 subjects in control group) and 18 months+ subgroup (*n* = 38, 20 subjects in LPN1115 group and 18 subjects in control group). No adverse events associated with the consumption of the tested LP N1115 were reported.

Stool consistency was evaluated using the Bristol stool scale (BSS). Only stool frequency and flatulence data could be analyzed, while the data for pain and bloating were considered negligible as it were not possible to conduct meaningful data analysis for these variables as variance. At the aspects of both stool consistency and flatulence, no significant differences were found between two groups (*p* > .05), and no changes for the entire sample overtime (*p* > .05). So were them in the 6–18 months subgroup (*p* > .05) (Figure 2b,c). However, the pattern of stool data showed that the LP N1115 group moved from soft to normal and the control group remained at soft. And it also showed the LP N1115 group remained stable while the control group experienced a spike in flatulence incidence in weeks 3–4, before stabilizing to a normal level similar to weeks 0–1.

### 3.2 Salivary cortisol

**FIGURE 3:**
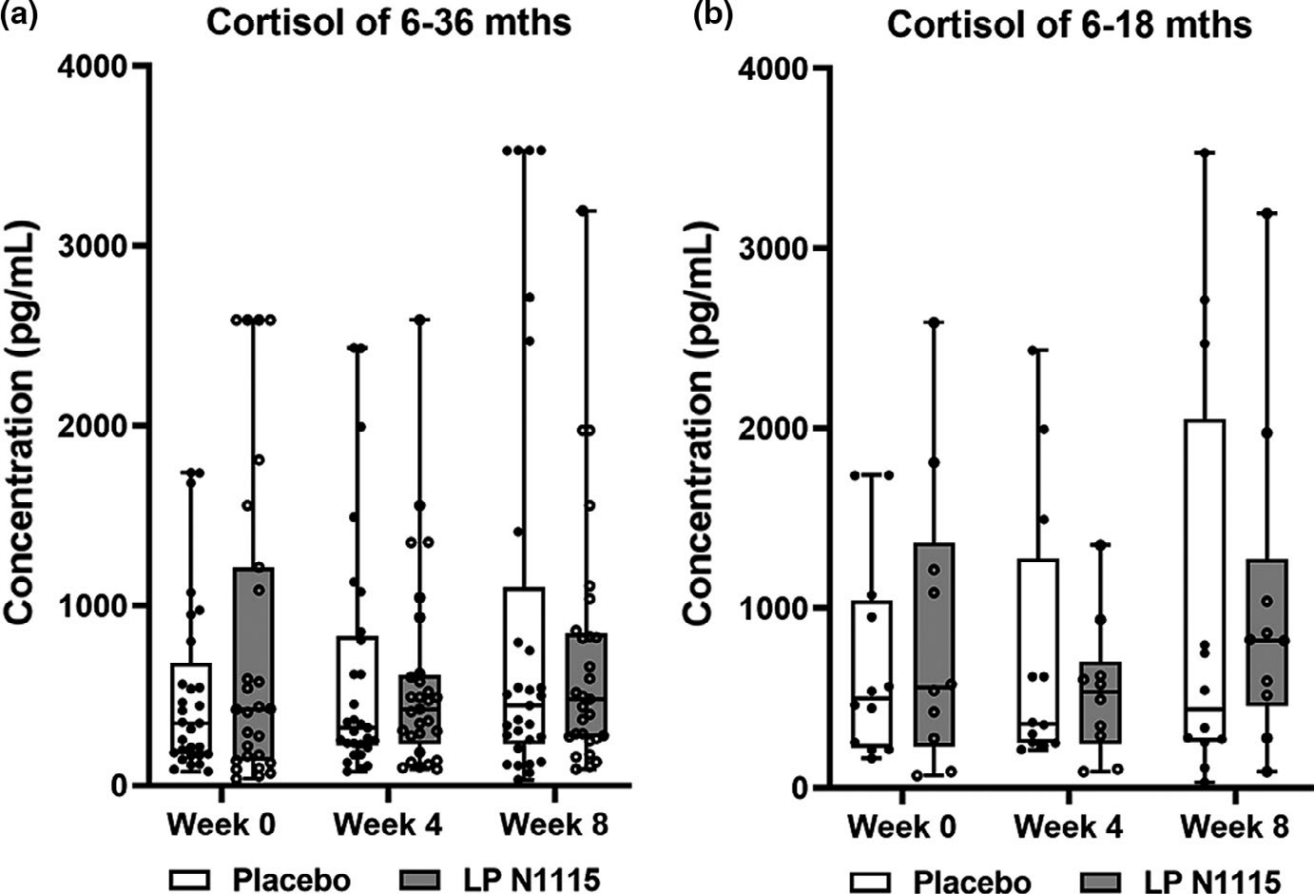
Salivary cortisol values (pg/ml) for both LP N1115 and placebo groups according to the different age groups. (a) Cortisol levels were assessed at baseline, week 4 and week 8 which showed no significant difference between two groups (*p* > .05). (b) Cortisol levels at the same three time points also showed no significant differences in the 6–18 months subgroup (*p* > .05). Hollow dots represent data of each subjects in the placebo group, and solid dots are data in LP N1115 group

Cortisol levels were assessed at baseline, week 4, and week 8 (Figure 3). Taking into account the whole populations, there were no changes in the entire sample overtime (*p* > .05) and no significant difference between two groups (*p* > .05). Likewise, there were no significant differences in the 6–18 months subgroup (*p* > .05).

### 3.3 Fecal microbiota composition

**FIGURE 4:**
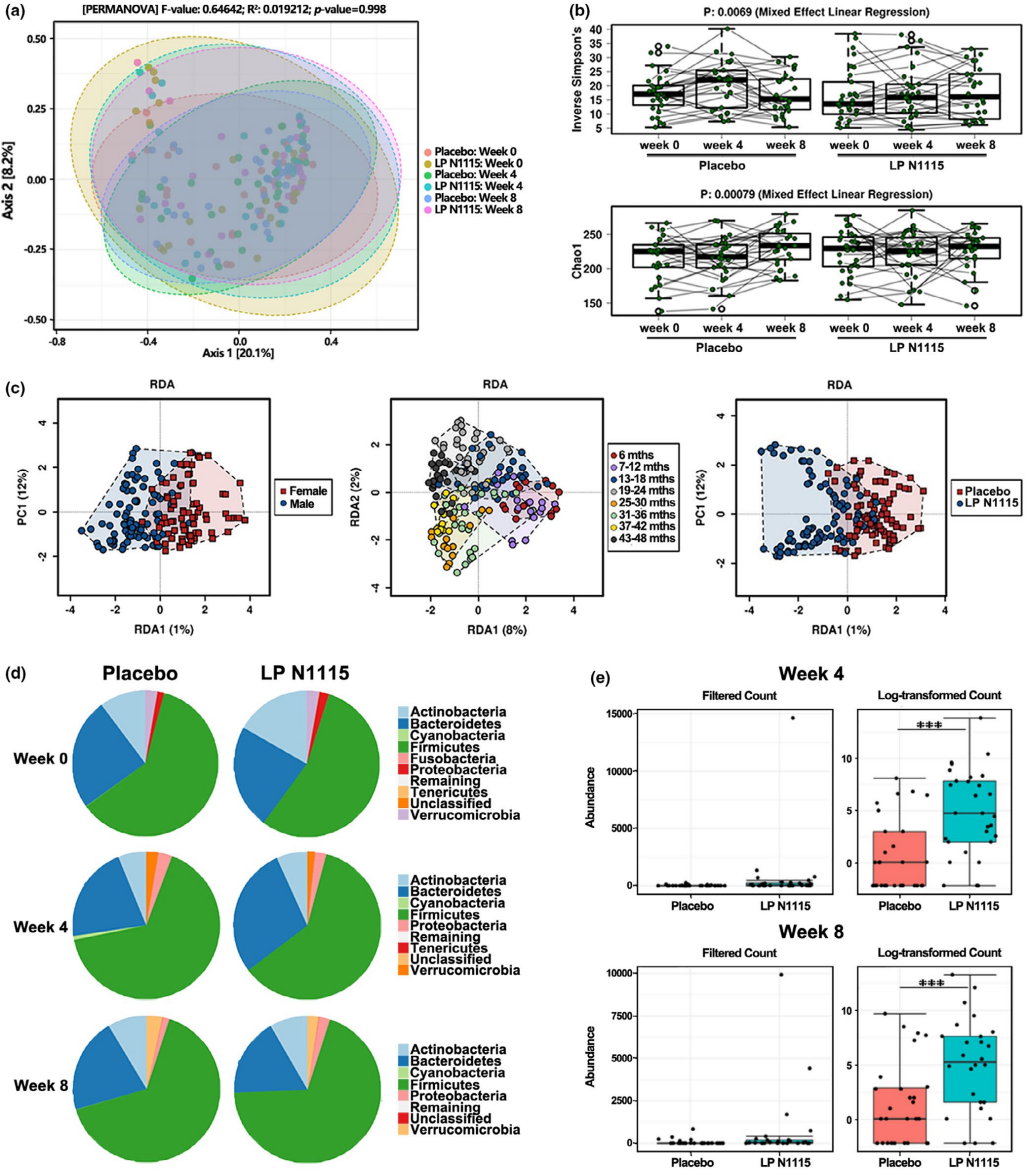
Fecal microbiota composition and diversity analysis of subjects. (a) PCoA represented by Bray‐Curtis Dissimilarity based on both LP N1115 and placebo groups over time (*p* > .05). (b) Inverse Simpson and Chao‐1 diversity using repeated measures based on OTUs between two groups over time. (c) RDA shows samples separate based on gender, age and treatment. Top 1000 OTU values. (d) Pie charts showing the relative abundance of the fecal microbiota at phylum level between the two groups at week 0, 4 and 8. (e) Differences in the relative abundance of *Lactobacillus* spp. in LP N1115 group at week 4 and week 8 was detected as significantly different using DESEq (*p* < .05)

As microbial changes in the infant gut are subject to variation and maturation overtime, age was stratified according to 6–18 and >18 months to illustrate the impact on microbial community and diversity across the time points (weeks 0, 4, and 8). Upon examination of the fecal microbiota, it was clear that age‐related differences have a strong influence on the separation of samples during the study. A principal coordinate analysis (PCoA) plot based on Bray–Curtis dissimilarity matrix at OTU level revealed a distinct separation between the samples (PERMANOVA *R* ^2^ = .07, *p* < .001) (Figure S1). When considering LP N1115 and placebo groups overtime, beta‐diversity was measured based on the Bray–Curtis dissimilarity matrix at OTU level and no visible separation or clustering of samples was evident (PERMANOVA *R* ^2^ = .01, *p* = .998) (Figure 4a). There were significant effects between two groups across the time points (*p* ≤ .01, *p* ≤ .01) by inverse Simpson and Chao‐1 alpha‐diversity measures (Figure 4b).

**FIGURE 5:**
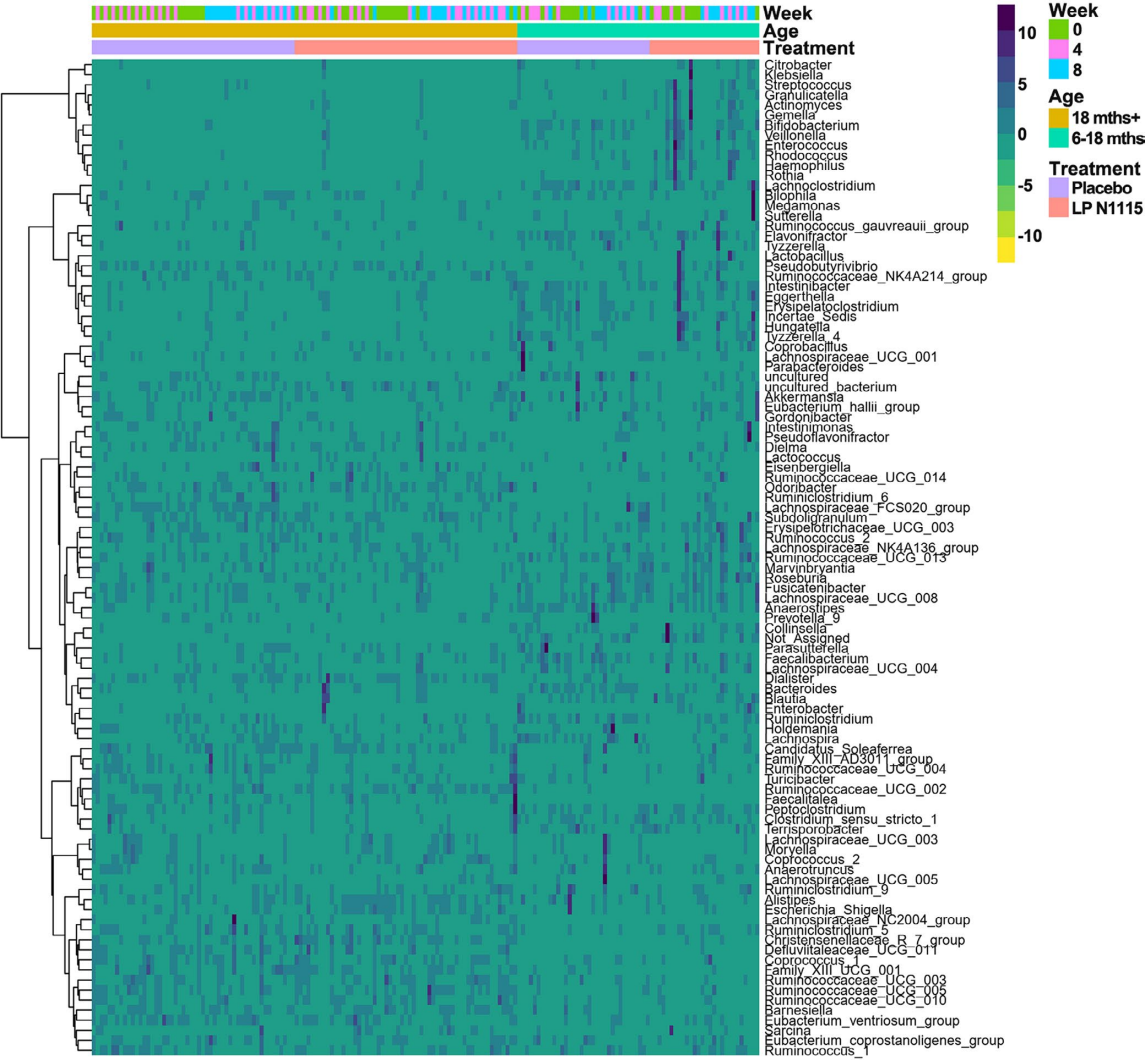
A Hierarchical clustering heat map of microbiota associated with treatment (including LP N1115 group in indy pink and the placebo group in lilac), age (including 6–18 months subgroup in cyan and 18 months+ subgroup in ginger) and time (including week 0 in olive green, week 4 in pink and week 8 in blue) based on Euclidean distance measure and Ward clustering algorithm. Values range from low (yellow) to high (navy)

A hierarchical clustering heatmap was used to investigate the relationships among age, treatment, and differentially abundant genera across all time points (Figure 5). The upper and mid sections on the right‐hand side of the heatmap show greater abundances of *Bifidobacterium*, *Klebsiella*, *Veillonella*, *Enterococcus*, *Rhodococcus*, *Actinomyces*, *Citrobacter*, *Blautia*, and *Clostridium_sensu_stricto_1* present in LP N1115 group and subjects aged 6–18 months. Bacteria such as these typically represent the core gut bacteria of C‐section delivered infants within the first year of life (Shao et al., 2019). In contrast, the mid and lower sections on the left‐hand side of the heatmap are dominated with high levels of *Ruminococcaceae*, *Lachnospiraceae,* and *Christensenellaceae spp*. that are known to be prevalent in the gut of young children between the ages of 3 and 4 years (Fouhy et al., 2019). In this instance, it was determined that gender, the age of subjects represented as years and months, and LP N1115 treatment explained the largest proportion of variance in terms of the microbiota (Figure 4c).

In total, 16 phyla were identified, of which, *Firmicutes* (62.49%) followed by *Bacteroidetes* (23.03%) and *Actinobacteria* (9.70%) dominated the microbiota over the course of the 8‐week intervention (Figure 4d). Although not statistically significant, Actinobacteria decreased in abundance from 17.70% at baseline to 7.00% by week 4 in LP N1115 group. Conversely, Bacteroidetes increased in abundance from 22.50% at baseline to 28.60% by week 4. In addition, there were a number of differentially abundant bacteria between LP N1115 and placebo groups at each time point and overtime (see Table S1 for all differentially abundant genera). In LP N1115 group, differences between baseline and week 4 included *Bifidobacterium*, *Gemella*, *Erysipelatoclostridium*, *Citrobacter*, *Enterococcus*, *Sarcina*, *Klebsiella*, *Clostridium_sensu_stricto_1,* and *Hungatella* (all *p* < .05). From baseline to week 8, *Gemella*, *Granulicatella*, *Bifidobacterium*, *Citrobacter*, and *Rothia* were significantly different. Lastly, *Prevotella_9* and *Megamonas* were significantly different between weeks 4 and 8. In the placebo group, *Megamonas* was significantly different between baseline and week 4 and between weeks 4 and 8. Interestingly, when comparing the two groups at each time point, a notably significant increase in the abundance of *Lactobacillus* at week 4 (0.44% vs. 0.01%, *p* < .05) and again at week 8 (0.40% vs. 0.04%, *p* < .05) was apparent in LP N1115 group in comparison to placebo (Figure 4e).

### 3.4 SCFAs

The most prevalent SCFAs detected in the fecal samples of all subjects at baseline and posttreatment (week 8) are shown in Table S2. There was a significant difference in propanoic acid levels between LP N1115 and placebo groups *F*[1, 56] = 7.93, *p* = .007. From baseline to postintervention, acetic acid (*F*[1, 57] = 7.11, *p* = .01) and butanoic acid (*F*[1, 56] = 4.93, *p* = .03) seemed to be significantly decreased in LP N1115 and placebo groups overtime. Considering the 6–18 months subgroup, significant differences in acetic acid (*F*[1, 20] = 6.20, *p* = .02), propanoic acid (*F*[1, 19] = 24.45, *p* < .0005), and butanoic acid (*F*[1, 19] = 7.49, *p* = .01) were observed between LP N1115 and placebo groups.

**FIGURE 6:**
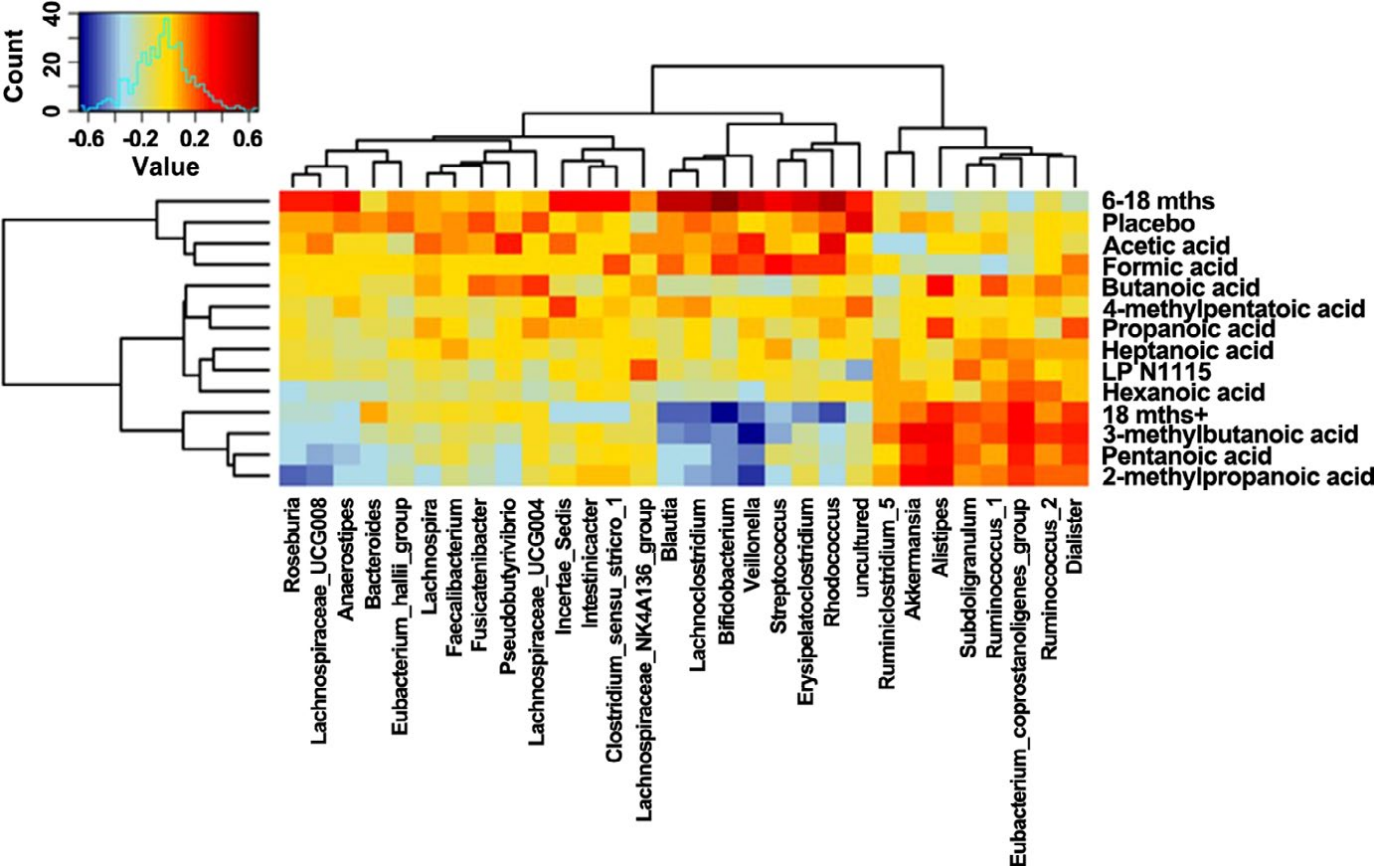
Spearman correlation heat map between SCFAs and relative abundances of fecal microbiota at the genus level from subjects based on both LP N1115 and placebo groups post‐intervention. Values range from low (blue) to high (red)

Spearman correlation analysis indicated clustering in relation to SCFA concentrations and the most abundant fecal microbiota postintervention (Figure 6). Following FDR correction, *Veillonella* appeared to be strongly, negatively correlated with 2‐methyl propanoic acid (*r* = −.58, *p* < .001), 3‐methyl butanoic acid (*r* = −.66, *p* < .001), and pentanoic acid (*r* = −.49, *p* < .01). On the other hand, genera including *Alistipes* and *Akkermansia* were found to be positively correlated with 2‐methyl propanoic acid (*r* = .38, *p* < .05; *r* = .32, *p* < .05) and 3‐methyl butanoic acid (*r* = .39, *p* < .05; *r* = .38, *p* < .05), respectively.

## 4 DISCUSSION

Extensive research in recent years has highlighted a global concern surrounding short‐ and long‐term health implications of C‐section delivery (Betrán et al., 2016). C‐section delivery is associated with an increased risk of health‐related disorders such as irritable bowel syndrome (IBS), allergy, obesity, and diabetes (Bager et al., 2008; Kuhle et al., 2015; Sevelsted et al., 2015). Of major concern is the perturbation of the gut microbiome as a result of C‐section, and some studies have shown that C‐section changes the normal microbial composition by reducing its diversity, such as decreasing the colonization of Bifidobacterium and *Bacteroides*, as well as increasing the number of *Clostridium difficile*, compared to vaginal delivery (Bäckhed et al., 2015; Penders et al., 2013). Clinical intervention studies using single‐ and multispecies probiotics, and in some cases supplemented with breast milk, have proven successful in exerting measurable effects on the gut microbiota of patients (Korpela et al., 2018; Quin et al., 2018). Thus, an opportunity to deliver potential therapies from an early age to treat and prevent debilitating diseases in later life represents a plausible avenue. Numerous studies have evaluated various probiotic strains, prebiotics, and synbiotics as a way of mediating immune regulation, microbial function, and diversity in C‐section delivered infants. Some of these interventions have proven successful in exerting measurable effects that help to restore the disrupted gut microbiota composition toward a microbial profile similar to vaginally delivered offspring (Chua et al., 2017; Garcia Rodenas et al., 2016; Hurkala et al., 2020; Korpela et al., 2018; Morais et al., 2020; Schultz et al., 2004). Given the promising clinical benefits associated with probiotics to date, the objective of this clinical trial was to evaluate the safety and efficacy of LP N1115 supplementation on gastrointestinal symptoms, salivary cortisol, gut microbiota, and SCFAs levels in a cohort of healthy, young children born by C‐section. A randomized, placebo‐controlled design was employed to minimize bias when comparing efficacy data during the intervention. The probiotic was found to be safe and well tolerated by all subjects throughout the study. Results showed that after 8 weeks of probiotic administration with a daily dose of 10^9^ CFU, gastrointestinal symptoms, cortisol levels, and fecal characteristics were generally similar for the placebo and LP N1115 groups.

When examining stool consistency as the primary efficacy variable, the results were similar between LP N1115 and placebo consumption overtime. This was not surprising given the fact that our study population was deemed as healthy; thus, this check was utilized to observe any adverse events related to the investigational product. Interestingly, when results from the additional subgroup analysis (6–18 months) were examined, a large effect size between LP N1115 and placebo groups overtime was found. Although the statistical comparison was nonsignificant, a trend in the data was observed with LP N1115 group moving from soft stools at baseline toward normal stools by week 8, whereas the placebo group moved toward softer stools by week 4 and returned to soft stools by week 8. Sample size was considered small in this subgroup, therefore, caution must be exercised when interpreting these findings. In some cases, the effects of LP N1115 are reported to be more pronounced in studies where gastrointestinal disorders are diagnosed. For example, a study by Urita et al. (2015) demonstrated that consumption of a fermented milk for 4 weeks containing *Bifidobacterium bifidum* YIT 10347 significantly improved gastrointestinal and psychological symptoms in patients aged 12–80 years. Similarly, Indrio and colleagues reported that prophylactic use of *Lactobacillus reuteri* DSM 17938 during the first 3 months of life significantly reduced the onset of functional gastrointestinal disorders, thus, reducing morbidity and costs associated with healthcare (Indrio et al., 2014).

No data analysis was performed for incidences of abdominal bloating and pain as minimal variance was reported in both groups. Moreover, this result may further strengthen the safety associated with LP N1115. Flatulence scores were similar between LP N1115 and placebo groups, albeit a trend toward significance was observed in LP N1115 group overtime. When analyzing the 6–18 months subgroup, a similar result was observed to that seen in the stool consistency data. Although not statistically significant, a large effect size was found and the pattern of data showed LP N1115 group remaining stable overtime, with the placebo group experiencing a spike in flatulence incidence by week 4, before decreasing to the same low level as baseline. Once again, these results should be interpreted cautiously considering both populations had low samples sizes.

Consistent with other studies, the gut microbial consortia at 6–18 months consisted of *Klebsiella*, *Veillonella*, *Citrob*acter, *Enterobacter*, *Enterococcus Clostridium,* and *Streptococcus,* whereas subjects older than 18 months were colonized by taxa such as *Dialister*, *Lachnospiraceae*, *Ruminococcaceae,* and *Christensenellaceae* (Azad et al., 2014; Bäckhed et al., 2015; Dominguez‐Bello et al., 2010; Fouhy et al., 2019; Hill et al., 2017). LP N1115 had no impact on the alpha‐ and beta‐diversity measures overtime or in comparison to the placebo at each time point. Conversely, there was a strong separation of the gut microbiota based on the age of the subjects when grouped as 6–18 and 18+ months. The only consistent change in relation to the gut microbiota overtime was a significant increase in *Lactobacillus* abundance at weeks 4 and 8 in LP N1115 group. This observation most likely reflects the transient colonization of LP N1115 within the gut microbiota of these subjects. Our results indicate that this increase in *Lactobacillus* is mainly due to the probiotic itself in the fecal samples. A similar result was also found in a study by Laursen et al. (2017), where two probiotics *Bifidobacterium animalis subsp. lactis* (BB‐12^®^) and *Lactobacillus rhamnosus* (LGG^®^) administered to infants aged 8–14 months for a period of 6 months did not significantly alter the gut microbial community and diversity compared to placebo, despite their detection in infant feces. Moreover, this further illustrates the importance of demonstrating efficacy of individual or multispecies probiotics in robust clinical trials.

Short‐chain fatty acids are end products produced from the fermentation of nondigestible dietary fibers and play fundamental roles in providing energy for colonocytes, acting as signaling molecules between the gut microbiota and host metabolism, and regulate immune function (den Besten et al., 2013). SCFAs such as acetic, propanoic, and butanoic acids were prevalent in both LP N1115 and placebo groups at baseline and postintervention (week 8). However, concentrations were subject to variation between the treatment groups and overtime. This may be partially explained by a number of factors. For example, information relating to the subjects' diet was not collected over the 8‐week intervention, thus, we cannot correlate any dietary variables to the levels of SCFAs seen in both groups. It is likely that LP N1115 intake for 8 weeks was not sufficient to alter the SCFA profiles in these infants/children. It is also important to note cross‐feeding interactions between preferential SCFA‐producing bacteria may have led to an accumulation of various products in vivo (Ríos‐Covián et al., 2016). In addition, SCFA concentrations were correlated with the most abundant fecal microbiota postintervention. SCFA‐producing bacteria including *Akkermansia* and *Alistipes* were positively correlated with 2‐methylpropanoic acid, pentanoic acid, and 3‐methylbutanoic acid, whereas *Veillonella*, *Bifidobacterium*, *Blautia*, *Lachnoclostridium,* and other genera were negatively associated with these SCFAs. Recent longitudinal studies have provided strong evidence of an increase in fecal butyrate concentration in infants between the ages of 3 and 12 months (Mueller et al., 2021; Nilsen et al., 2020). Interestingly, the former study observed a significant increase in butyrate production at 3 months but not at 12 months with respect to C‐section delivery.

In light of the above, this study has some limitations. Firstly, due to the exploratory nature of this study, sample size was limited and not statistically powered to conclude on any clinical health‐related end points. Secondly, a washout period and a longer follow‐up would have been useful to evaluate the differences between the treatment groups more closely. Thirdly, additional factors such as aspects of genetic influence, lifestyle, antibiotic usage, siblings, or household pets which may have an effect on gut microbiota were not studied. In addition, the preliminary findings of this study may warrant a larger clinical trial to elucidate the possible role of LP N1115 administration with regards to a younger cohort.

## CONFLICT OF INTEREST

No conflict of interest stated from authors.

## AUTHOR CONTRIBUTION

**Shijie Wang:** Conceptualization (equal); Project administration (equal); Writing‐original draft (equal). **Yiping Xun:** Conceptualization (equal); Writing‐original draft (equal). **Grace Ahern:** Investigation (equal); Writing‐original draft (equal). **Lili Feng:** Project administration (equal); Validation (equal). **Dong Zhang:** Software (equal); Visualization (equal). **Yuling Xue:** Project administration (equal); Validation (equal). **Reynolds Paul Ross:** Investigation (equal); Methodology (equal). **Andrea Doolan:** Project administration (equal). **Catherine Stanton:** Methodology (equal); Validation (equal). **Hong Zhu:** Conceptualization (equal); Project administration (equal).

## ETHICAL APPROVAL

The study's protocols and procedures were ethically reviewed and approved by the Clinical Research Ethics Committee of the Cork Teaching Hospitals (CREC, Ireland), and written, informed consent was obtained from all parents/guardians on behalf of the child in the current project. The trial was conducted in accordance with the ethical principles set forth in the current version of the Declaration of Helsinki.

## Supporting information

Supplementary Material

## References

[fsn32533-bib-0001] Arrieta, M.‐C. , Stiemsma, L. T. , Amenyogbe, N. , Brown, E. M. , & Finlay, B. (2014). The intestinal microbiome in early life: Health and disease. Frontiers in Immunology, 5, 427. 10.3389/fimmu.2014.00427 25250028 PMC4155789

[fsn32533-bib-0002] Azad, M. B. , Konya, T. , Guttman, D. S. , Field, C. J. , Chari, R. S. , Sears, M. R. , Becker, A. B. , Scott, J. A. , & Kozyrskyj, A. L. (2014). Impact of cesarean section delivery and breastfeeding on infant gut microbiota at one year of age. Allergy, Asthma, and Clinical Immunology, 10(Suppl 1), A24. 10.1186/1710-1492-10-S1-A24

[fsn32533-bib-0003] Azad, M. B. , Konya, T. , Persaud, R. R. , Guttman, D. S. , Chari, R. S. , Field, C. J. , Sears, M. R. , Mandhane, P. J. , Turvey, S. E. , Subbarao, P. , Becker, A. B. , Scott, J. A. , & Kozyrskyj, A. L. (2016). Impact of maternal intrapartum antibiotics, method of birth and breastfeeding on gut microbiota during the first year of life: A prospective cohort study. BJOG: An International Journal of Obstetrics & Gynaecology, 123(6), 983–993. 10.1111/1471-0528.13601 26412384

[fsn32533-bib-0004] Bäckhed, F. , Roswall, J. , Peng, Y. , Feng, Q. , Jia, H. , Kovatcheva‐Datchary, P. , Li, Y. , Xia, Y. , Xie, H. , Zhong, H. , Khan, M. T. , Zhang, J. , Li, J. , Xiao, L. , Al‐Aama, J. , Zhang, D. , Lee, Y. S. , Kotowska, D. , Colding, C. , … Wang, J. (2015). Dynamics and stabilization of the human gut microbiome during the first year of life. Cell Host & Microbe, 17(5), 690–703. 10.1016/j.chom.2015.04.004 25974306

[fsn32533-bib-0005] Bager, P. , Simonsen, J. , Nielsen, N. M. , & Frisch, M. (2012). Cesarean section and offspring's risk of inflammatory bowel disease: A national cohort study. Inflammatory Bowel Diseases, 18(5), 857–862. 21739532 10.1002/ibd.21805

[fsn32533-bib-0006] Bager, P. , Wohlfahrt, J. , & Westergaard, T. (2008). Caesarean delivery and risk of atopy and allergic disease: Meta‐analyses. Clinical & Experimental Allergy, 38(4), 634–642. 18266879 10.1111/j.1365-2222.2008.02939.x

[fsn32533-bib-0007] Betrán, A. P. , Ye, J. , Moller, A.‐B. , Zhang, J. , Gülmezoglu, A. M. , & Torloni, M. R. (2016). The increasing trend in caesarean section rates: Global, regional and national estimates: 1990–2014. PLoS One, 11(2), e0148343. 10.1371/journal.pone.0148343 26849801 PMC4743929

[fsn32533-bib-0008] Bezirtzoglou, E. , Tsiotsias, A. , & Welling, G. W. (2011). Microbiota profile in feces of breast‐ and formula‐fed newborns by using fluorescence in situ hybridization (FISH). Anaerobe, 17(6), 478–482. 10.1016/j.anaerobe.2011.03.009 21497661

[fsn32533-bib-0009] Cao, Y. , Zhang, J. , Zheng, Z. , Mei, X. , Wang, S. , Zhu, H. , & Yang, Z. (2018). Therapeutic effect of *Lactobacillus paracasei* N1115 fermented milk in constipated mice. Shipin Kexue/Food Science, 39(1), 185–191.

[fsn32533-bib-0010] Cardwell, C. R. , Stene, L. C. , Joner, G. , Cinek, O. , Svensson, J. , Goldacre, M. J. , & Stoyanov, D. (2008). Caesarean section is associated with an increased risk of childhood‐onset type 1 diabetes mellitus: A meta‐analysis of observational studies. Springer. 10.1007/s00125-008-0941-z 18292986

[fsn32533-bib-0011] Chua, M. C. , Ben‐Amor, K. , Lay, C. , Goh, A. E. N. , Chiang, W. C. , Rao, R. , Chew, C. , Chaithongwongwatthana, S. , Khemapech, N. , Knol, J. , & Chongsrisawat, V. (2017). Effect of synbiotic on the gut microbiota of cesarean delivered infants: A randomized, double‐blind, multicenter study. Journal of Pediatric Gastroenterology and Nutrition, 65(1), 102–106. 10.1097/mpg.0000000000001623 28644357

[fsn32533-bib-0012] Clemente, J. C. , Ursell, L. K. , Parfrey, L. W. , & Knight, R. (2012). The impact of the gut microbiota on human health: An integrative view. Cell, 148(6), 1258–1270. 10.1016/j.cell.2012.01.035 22424233 PMC5050011

[fsn32533-bib-0013] Collado, M. C. , Isolauri, E. , Laitinen, K. , & Salminen, S. (2010). Effect of mother's weight on infant's microbiota acquisition, composition, and activity during early infancy: A prospective follow‐up study initiated in early pregnancy. The American Journal of Clinical Nutrition, 92(5), 1023–1030. 10.3945/ajcn.2010.29877 20844065

[fsn32533-bib-0014] den Besten, G. , van Eunen, K. , Groen, A. K. , Venema, K. , Reijngoud, D.‐J. , & Bakker, B. M. (2013). The role of short‐chain fatty acids in the interplay between diet, gut microbiota, and host energy metabolism. Journal of Lipid Research, 54(9), 2325–2340. 10.1194/jlr.R036012 23821742 PMC3735932

[fsn32533-bib-0015] Dhariwal, A. , Chong, J. , Habib, S. , King, I. L. , Agellon, L. B. , & Xia, J. (2017). MicrobiomeAnalyst: A web‐based tool for comprehensive statistical, visual and meta‐analysis of microbiome data. Nucleic Acids Research, 45(W1), W180–W188. 10.1093/nar/gkx295 28449106 PMC5570177

[fsn32533-bib-0016] Di Cerbo, A. , Palmieri, B. , Aponte, M. , Morales‐Medina, J. C. , & Iannitti, T. (2016). Mechanisms and therapeutic effectiveness of lactobacilli. Journal of Clinical Pathology, 69(3), 187. 10.1136/jclinpath-2015-202976 26578541 PMC4789713

[fsn32533-bib-0017] Dominguez‐Bello, M. G. , Costello, E. K. , Contreras, M. , Magris, M. , Hidalgo, G. , Fierer, N. , & Knight, R. (2010). Delivery mode shapes the acquisition and structure of the initial microbiota across multiple body habitats in newborns. Proceedings of the National Academy of Sciences of the United States of America, 107(26), 11971–11975. 10.1073/pnas.1002601107 20566857 PMC2900693

[fsn32533-bib-0018] Edgar, R. C. (2010). Search and clustering orders of magnitude faster than BLAST. Bioinformatics, 26(19), 2460–2461. 10.1093/bioinformatics/btq461 20709691

[fsn32533-bib-0019] Fouhy, F. , Watkins, C. , Hill, C. J. , O'Shea, C.‐A. , Nagle, B. , Dempsey, E. M. , O'Toole, P. W. , Ross, R. P. , Ryan, C. A. , & Stanton, C. (2019). Perinatal factors affect the gut microbiota up to four years after birth. Nature Communications, 10(1), 1517. 10.1038/s41467-019-09252-4 PMC6447568 30944304

[fsn32533-bib-0020] Garcia Rodenas, C. L. , Lepage, M. , Ngom‐Bru, C. , Fotiou, A. , Papagaroufalis, K. , & Berger, B. (2016). Effect of formula containing *Lactobacillus reuteri* DSM 17938 on fecal microbiota of infants born by cesarean‐section. Journal of Pediatric Gastroenterology and Nutrition, 63(6), 681–687. 10.1097/mpg.0000000000001198 27035371

[fsn32533-bib-0021] Guo, Y. , Shi, L. , He, M. , Zhang, L. , Shen, X. , Zhu, H. , & Huang, C. (2016). Prevention of acute upper tract infections on the consumption of fermented dairy product in the middle ‐ Aged and the elderly. The Medical Journal of the Present Clinical, 43(5), 810–813.

[fsn32533-bib-0022] Gupta, M. , & Saini, V. (2018). Caesarean section: Mortality and morbidity. Risk, 2, 53.

[fsn32533-bib-0023] Hill, C. J. , Lynch, D. B. , Murphy, K. , Ulaszewska, M. , Jeffery, I. B. , O'Shea, C. A. , Watkins, C. , Dempsey, E. , Mattivi, F. , Tuohy, K. , Ross, R. P. , Ryan, C. A. , O'Toole, P. W. , & Stanton, C. (2017). Evolution of gut microbiota composition from birth to 24 weeks in the INFANTMET Cohort. Microbiome, 5(1), 4. 10.1186/s40168-016-0213-y 28095889 PMC5240274

[fsn32533-bib-0024] Hurkala, J. , Lauterbach, R. , Radziszewska, R. , Strus, M. , & Heczko, P. (2020). Effect of a short‐time probiotic supplementation on the abundance of the main constituents of the gut microbiota of term newborns delivered by cesarean section‐A randomized, prospective, controlled clinical trial. Nutrients, 12(10), 3128. 10.3390/nu12103128 PMC7602088 33066338

[fsn32533-bib-0025] Indrio, F. , Di Mauro, A. , Riezzo, G. , Civardi, E. , Intini, C. , Corvaglia, L. , Ballardini, E. , Bisceglia, M. , Cinquetti, M. , Brazzoduro, E. , Del Vecchio, A. , Tafuri, S. , & Francavilla, R. (2014). Prophylactic use of a probiotic in the prevention of colic, regurgitation, and functional constipation: A randomized clinical trial. JAMA Pediatrics, 168(3), 228–233. 10.1001/jamapediatrics.2013.4367 24424513

[fsn32533-bib-0026] Korpela, K. , Salonen, A. , Vepsäläinen, O. , Suomalainen, M. , Kolmeder, C. , Varjosalo, M. , Miettinen, S. , Kukkonen, K. , Savilahti, E. , Kuitunen, M. , & de Vos, W. M. (2018). Probiotic supplementation restores normal microbiota composition and function in antibiotic‐treated and in caesarean‐born infants. Microbiome, 6(1), 182. 10.1186/s40168-018-0567-4 30326954 PMC6192119

[fsn32533-bib-0027] Kuhle, S. , Tong, O. , & Woolcott, C. (2015). Association between caesarean section and childhood obesity: A systematic review and meta‐analysis. Obesity Reviews, 16(4), 295–303. 10.1111/obr.12267 25752886

[fsn32533-bib-0028] Laursen, M. F. , Laursen, R. P. , Larnkjær, A. , Michaelsen, K. F. , Bahl, M. I. , & Licht, T. R. (2017). Administration of two probiotic strains during early childhood does not affect the endogenous gut microbiota composition despite probiotic proliferation. BMC Microbiology, 17(1), 175. 10.1186/s12866-017-1090-7 28818050 PMC5561568

[fsn32533-bib-0029] Mårild, K. , Stephansson, O. , Montgomery, S. , Murray, J. A. , & Ludvigsson, J. F. (2012). Pregnancy outcome and risk of celiac disease in offspring: A nationwide case‐control study. Gastroenterology, 142(1), 39–45.e33. 10.1053/j.gastro.2011.09.047 21995948 PMC3244504

[fsn32533-bib-0030] Mathur, H. , Beresford, T. P. , & Cotter, P. D. (2020). Health benefits of lactic acid bacteria (LAB) fermentates. Nutrients, 12(6), 1679. 10.3390/nu12061679 PMC7352953 32512787

[fsn32533-bib-0031] Milani, C. , Duranti, S. , Bottacini, F. , Casey, E. , Turroni, F. , Mahony, J. , Belzer, C. , Delgado Palacio, S. , Arboleya Montes, S. , Mancabelli, L. , Lugli, G. A. , Rodriguez, J. M. , Bode, L. , de Vos, W. , Gueimonde, M. , Margolles, A. , van Sinderen, D. , & Ventura, M. (2017). The first microbial colonizers of the human gut: Composition, activities, and health implications of the infant gut microbiota. Microbiology and Molecular Biology Reviews, 81(4), e00036‐17. 10.1128/MMBR.00036-17 PMC5706746 29118049

[fsn32533-bib-0032] Morais, L. H. , Golubeva, A. V. , Moloney, G. M. , Moya‐Pérez, A. , Ventura‐Silva, A. P. , Arboleya, S. , Bastiaanssen, T. F. S. , O'Sullivan, O. , Rea, K. , Borre, Y. , Scott, K. A. , Patterson, E. , Cherry, P. , Stilling, R. , Hoban, A. E. , El Aidy, S. , Sequeira, A. M. , Beers, S. , Moloney, R. D. , … Cryan, J. F. (2020). Enduring behavioral effects induced by birth by caesarean section in the mouse. Current Biology, 30(19), 3761–3774.e3766. 10.1016/j.cub.2020.07.044 32822606

[fsn32533-bib-0033] Mueller, N. , Differding, M. , Østbye, T. , Hoyo, C. , & Benjamin‐Neelon, S. (2021). Association of birth mode of delivery with infant faecal microbiota, potential pathobionts, and short chain fatty acids: A longitudinal study over the first year of life. BJOG: An International Journal of Obstetrics & Gynaecology, 128(8), 1293–1303. 10.1111/1471-0528.16633 33338292 PMC8211907

[fsn32533-bib-0034] Neu, J. , & Rushing, J. (2011). Cesarean versus vaginal delivery: Long‐term infant outcomes and the hygiene hypothesis. Clinics in Perinatology, 38(2), 321–331. 10.1016/j.clp.2011.03.008 21645799 PMC3110651

[fsn32533-bib-0035] Nilsen, M. , Madelen Saunders, C. , Leena Angell, I. , Arntzen, M. Ø. , Lødrup Carlsen, K. C. , Carlsen, K.‐H. , Haugen, G. , Heldal Hagen, L. , Carlsen, M. H. , Hedlin, G. , Monceyron Jonassen, C. , Nordlund, B. , Maria Rehbinder, E. , Skjerven, H. O. , Snipen, L. , Cathrine Staff, A. , Vettukattil, R. , & Rudi, K. (2020). Butyrate levels in the transition from an infant‐to an adult‐like gut microbiota correlate with bacterial networks associated with *Eubacterium rectale* and *Ruminococcus gnavus* . Genes, 11(11), 1245. 10.3390/genes11111245 PMC7690385 33105702

[fsn32533-bib-0036] Penders, J. , Gerhold, K. , Stobberingh, E. E. , Thijs, C. , Zimmermann, K. , Lau, S. , & Hamelmann, E. (2013). Establishment of the intestinal microbiota and its role for atopic dermatitis in early childhood. The Journal of Allergy and Clinical Immunology, 132(3), 601–607.e8. 10.1016/j.jaci.2013.05.043 23900058

[fsn32533-bib-0037] Pu, F. , Guo, Y. , Li, M. , Zhu, H. , Wang, S. , Shen, X. , & He, F. (2017). Yogurt supplemented with probiotics can protect the healthy elderly from respiratory infections: A randomized controlled open‐label trial. Clinical Interventions in Aging, 12, 1223–1231. 10.2147/cia.S141518 28848330 PMC5557113

[fsn32533-bib-0048] Quin C. , Estaki M. , Vollman D. M. , Barnett J. A. , Gill S. K. , & Gibson D. L. (2018). Probiotic supplementation and associated infant gut microbiome and health: a cautionary retrospective clinical comparison. Scientific Reports, 8(1). 10.1038/s41598-018-26423-3 PMC5974413 29844409

[fsn32533-bib-0038] Ríos‐Covián, D. , Ruas‐Madiedo, P. , Margolles, A. , Gueimonde, M. , de Los Reyes‐Gavilán, C. G. , & Salazar, N. (2016). Intestinal short chain fatty acids and their link with diet and human health. Frontiers in Microbiology, 7, 185. 10.3389/fmicb.2016.00185 26925050 PMC4756104

[fsn32533-bib-0039] Rodríguez, J. M. , Murphy, K. , Stanton, C. , Ross, R. P. , Kober, O. I. , Juge, N. , Avershina, E. , Rudi, K. , Narbad, A. , Jenmalm, M. C. , Marchesi, J. R. , & Collado, M. C. (2015). The composition of the gut microbiota throughout life, with an emphasis on early life. Microbial Ecology in Health and Disease, 26(1), 26050. 10.3402/mehd.v26.26050 25651996 PMC4315782

[fsn32533-bib-0040] Schultz, M. , Göttl, C. , Young, R. J. , Iwen, P. , & Vanderhoof, J. A. (2004). Administration of oral probiotic bacteria to pregnant women causes temporary infantile colonization. Journal of Pediatric Gastroenterology and Nutrition, 38(3), 293–297. 10.1097/00005176-200403000-00012 15076629

[fsn32533-bib-0041] Sevelsted, A. , Stokholm, J. , Bønnelykke, K. , & Bisgaard, H. (2015). Cesarean section and chronic immune disorders. Pediatrics, 135(1), e92–e98. 10.1542/peds.2014-0596 25452656

[fsn32533-bib-0042] Shao, Y. , Forster, S. C. , Tsaliki, E. , Vervier, K. , Strang, A. , Simpson, N. , Kumar, N. , Stares, M. D. , Rodger, A. , Brocklehurst, P. , Field, N. , & Lawley, T. D. (2019). Stunted microbiota and opportunistic pathogen colonization in caesarean‐section birth. Nature, 574(7776), 117–121. 10.1038/s41586-019-1560-1 31534227 PMC6894937

[fsn32533-bib-0043] Shin, H. , Pei, Z. , Martinez, K. A. , Rivera‐Vinas, J. I. , Mendez, K. , Cavallin, H. , & Dominguez‐Bello, M. G. (2015). The first microbial environment of infants born by C‐section: The operating room microbes. Microbiome, 3(1), 59. 10.1186/s40168-015-0126-1 26620712 PMC4665759

[fsn32533-bib-0044] Tapiainen, T. , Koivusaari, P. , Brinkac, L. , Lorenzi, H. A. , Salo, J. , Renko, M. , Pruikkonen, H. , Pokka, T. , Li, W. , Nelson, K. , Pirttilä, A. M. , & Tejesvi, M. V. (2019). Impact of intrapartum and postnatal antibiotics on the gut microbiome and emergence of antimicrobial resistance in infants. Scientific Reports, 9(1), 10635. 10.1038/s41598-019-46964-5 31337807 PMC6650395

[fsn32533-bib-0045] Urita, Y. , Goto, M. , Watanabe, T. , Matsuzaki, M. , Gomi, A. , Kano, M. , & Kaneko, H. (2015). Continuous consumption of fermented milk containing Bifidobacterium bifidum YIT 10347 improves gastrointestinal and psychological symptoms in patients with functional gastrointestinal disorders. Bioscience of Microbiota, Food and Health, 34(2), 37–44. 10.12938/bmfh.2014-017 PMC4405396 25918671

[fsn32533-bib-0046] Wang, S. , Yan, F. , He, F. , & Zhu, H. (2016). Effects of *Lactobacillus paracasei* N1115 on intestinal development in neonatal mice. Acta Nutrimenta Sinica, 38(1), 71–74.

[fsn32533-bib-0047] Yu Zhongtang , & Morrison Mark (2004). Improved extraction of PCR‐quality community DNA from digesta and fecal samples. BioTechniques, 36(5), 808–812. 10.2144/04365st04 15152600

