# Supplementary material for: A randomized, double blind, parallel, placebo‐controlled study to investigate the efficacy of Lactobacillus paracasei N1115 in gut development of young children
Source: Food Sci Nutr. 2021 Sep 1;9(11):6020–30. doi: 10.1002/fsn3.2533 (PMC8565250; doi:10.1002/fsn3.2533)
Supplement: Supplementary file 1 — Supplementary Material [file FSN3-9-6020-s001.docx]

#### Supplementary Information

**A randomized, double-blinded, parallel, placebo-controlled study to investigate the efficacy of *Lactobacillus paracasei* N1115 in gut development of young children**

**Shijie Wang, Yiping Xun,** **Grace J. Ahern, Lili Feng, Dong Zhang, Yuling Xue, R. Paul Ross,** **Andrea M. Doolan, Catherine Stanton, Hong Zhu**


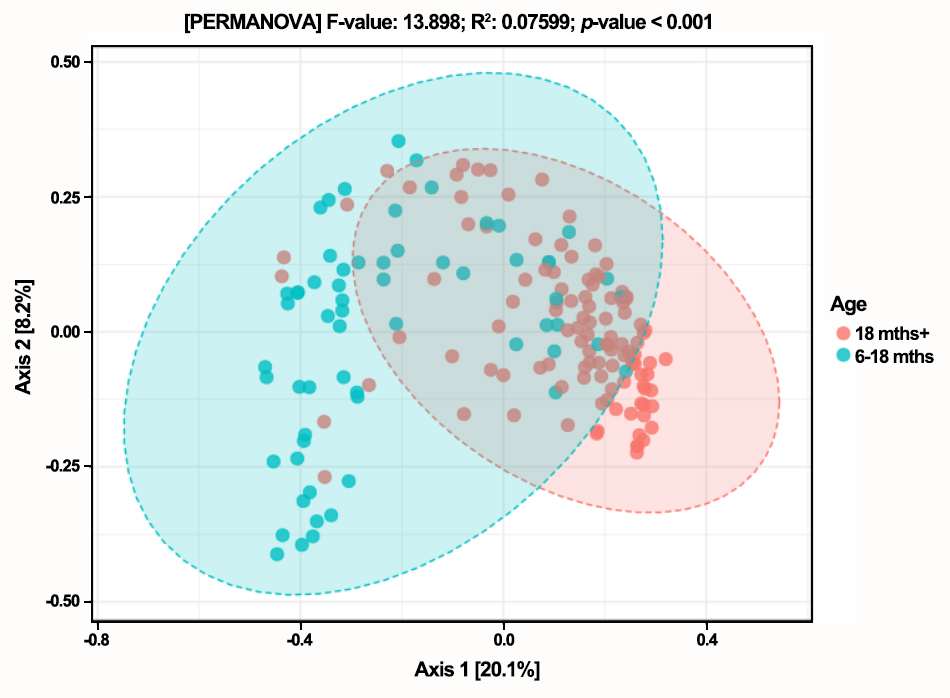


**FIGURE S1** PCoA based on Bray-Curtis dissimilarity matrix at OTU level revealed a distinct separation between the samples based on age of 6-18 months and > 18 months. The red points represent datas from subjects aged above 18 months, and blue ones are from subjects aged between 6 to18 months.

**TABLE S1** Differentially abundant genera between the probiotic and placebo groups over time and between each time point. Log2FC = log2 fold change, lfcSE = log fold change standard error.

|  | Baseline probiotic vs. week 4 probiotic | | | |
| --- | --- | --- | --- | --- |
|  | **Log2FC** | **lfcSE** | ***p*-value** | **FDR** |
| *Bifidobacterium* | -4.0886 | 0.73104 | 2.23E-08 | 2.21E-06 |
| *Gemella* | -4.783 | 0.88986 | 7.66E-08 | 3.79E-06 |
| *Erysipelatoclostridium* | -2.4413 | 0.60216 | 5.03E-05 | 0.0016598 |
| *Citrobacter* | -3.9346 | 1.0523 | 0.00018473 | 0.0045721 |
| *Enterococcus* | -3.8304 | 1.072 | 0.00035254 | 0.0069803 |
| *Sarcina* | 3.7493 | 1.1438 | 0.0010454 | 0.017249 |
| *Klebsiella* | -3.5699 | 1.1249 | 0.0015058 | 0.021296 |
| *Clostridium_sensu_stricto_1* | 1.5494 | 0.50503 | 0.0021561 | 0.026682 |
| *Hungatella* | -1.6088 | 0.54488 | 0.0031507 | 0.034657 |
|  | Baseline probiotic vs. week 8 probiotic | | | |
| *Gemella* | -5.162 | 0.90699 | 1.26E-08 | 1.24E-06 |
| *Granulicatella* | -3.0705 | 0.73897 | 3.25E-05 | 0.0015936 |
| *Bifidobacterium* | -3.0328 | 0.77513 | 9.13E-05 | 0.0029828 |
| *Citrobacter* | -4.2573 | 1.1828 | 0.000319 | 0.0078154 |
| *Rothia* | -3.0753 | 0.87266 | 0.00042502 | 0.0083303 |
|  | Week 4 probiotic vs. week 8 probiotic | | | |
| *Prevotella_9* | 3.9212 | 0.98465 | 6.82E-05 | 0.005273 |
| *Megamonas* | 4.5282 | 1.1685 | 0.00010653 | 0.005273 |
|  | Baseline placebo vs. week 4 placebo | | | |
| *Megamonas* | -5.9985 | 1.6164 | 0.00020641 | 0.020435 |
|  | Baseline placebo vs. week 8 placebo | | | |
| *Enterobacter* | -4.2236 | 0.9544 | 9.63E-06 | 0.00095313 |
| *Citrobacter* | -3.4484 | 0.92229 | 0.0001848 | 0.0091478 |
| *Terrisporobacter* | -1.9834 | 0.56857 | 0.00048597 | 0.016037 |
| *Moryella* | -3.2569 | 1.0237 | 0.0014645 | 0.036246 |
|  | Week 4 placebo vs. week 8 placebo | | | |
| *Megamonas* | 5.4468 | 0.86549 | 3.11E-10 | 3.08E-08 |
|  | Baseline placebo vs. baseline probiotic | | | |
| *Klebsiella* | 6.7663 | 1.0713 | 2.68E-10 | 2.66E-08 |
| *Gemella* | 4.2298 | 0.79081 | 8.86E-08 | 4.39E-06 |
| *uncultured* | -1.7255 | 0.39421 | 1.20E-05 | 0.00039662 |
| *Enterococcus* | 3.9424 | 0.9278 | 2.15E-05 | 0.00053102 |
| *Actinomyces* | 2.3748 | 0.64703 | 0.00024227 | 0.004797 |
| *Prevotella_9* | -3.6631 | 1.0739 | 0.00064725 | 0.01068 |
| *Rothia* | 2.6991 | 0.82032 | 0.0010009 | 0.013891 |
| *Terrisporobacter* | -1.6349 | 0.50182 | 0.0011225 | 0.013891 |
| *Veillonella* | 2.74 | 0.89815 | 0.0022826 | 0.023279 |
| *Citrobacter* | 3.0238 | 0.99408 | 0.0023514 | 0.023279 |
| *Granulicatella* | 2.0534 | 0.6914 | 0.0029795 | 0.026815 |
| *Lactococcus* | 2.4289 | 0.87414 | 0.0054595 | 0.044232 |
| *Sarcina* | 3.1145 | 1.1298 | 0.0058403 | 0.044232 |
| *Streptococcus* | 1.4972 | 0.54761 | 0.006255 | 0.044232 |
|  | Week 4 placebo vs. week 4 probiotic | | | |
| *Klebsiella* | 5.8464 | 1.1266 | 2.11E-07 | 2.09E-05 |
| *Megamonas* | 4.8524 | 1.0273 | 2.32E-06 | 0.00011481 |
| *uncultured* | -1.5915 | 0.39002 | 4.49E-05 | 0.001482 |
| *Lactobacillus* | 3.2432 | 0.85283 | 0.00014306 | 0.0035408 |
| *Erysipelatoclostridium* | -1.6814 | 0.5089 | 0.00095331 | 0.018876 |
| *Veillonella* | 2.2014 | 0.71155 | 0.0019761 | 0.032606 |
| *Ruminococcaceae_UCG_014* | 2.9933 | 0.98773 | 0.0024416 | 0.034531 |
|  | Week 8 placebo vs. week 8 probiotic | | | |
| *Lactobacillus* | 5.1518 | 0.84903 | 1.30E-09 | 1.28E-07 |
| *Klebsiella* | 6.6078 | 1.28 | 2.44E-07 | 1.21E-05 |
| *Enterobacter* | 5.4124 | 1.1793 | 4.45E-06 | 0.00014668 |
| *Megamonas* | 3.8928 | 1.037 | 0.00017413 | 0.0043096 |
| *uncultured* | -1.3842 | 0.39837 | 0.0005115 | 0.010128 |

**TABLE S2** Comparisons of fecal SCFAs by treatment (LP N1115 and placebo), study population (all subjects and 6-18 mths subgroup) and time point (baseline and post-intervention).

|  |  |  | **Placebo** |  | ***L. paracasei N1115*** |
| --- | --- | --- | --- | --- | --- |
|  |  |  |  |  |  |
|  | mM | n |  | n |  |
|  |  |  |  |  |  |
| Baseline (week 0) |  |  |  |  |  |
| All subjects | Acetic acid | 30 | 32.01 ± 10.60** | 29 | 29.08 ± 11.73** |
|  | Propanoic acid | 30 | 10.33 ± 5.47* | 28 | 8.03 ± 6.51* |
|  | 2-methyl-propanoic acid | 28 | 0.824 ± 0.360 | 26 | 0.769 ± 0.397 |
|  | Butanoic acid | 30 | 8.85 ± 5.60** | 28 | 8.60 ± 6.11** |
|  | 3-methyl-butanoic acid | 28 | 0.351 ± 0.196 | 24 | 0.338 ± 0.182 |
|  | Pentanoic acid | 22 | 0.771 ± 0.562 | 22 | 0.790 ± 0.550 |
|  |  |  |  |  |  |
| 6-18 mths | Acetic acid | 12 | 35.63 ± 12.25* | 10 | 27.36 ± 7.61* |
|  | Propanoic acid | 12 | 11.13 ± 7.42* | 9 | 4.32 ± 1.80* |
|  | 2-methyl-propanoic acid | 11 | 0.711 ± 0.343 | 7 | 0.556 ± 0.227 |
|  | Butanoic acid | 12 | 7.83 ± 4.77* | 9 | 4.17 ± 2.37* |
|  | 3-methyl-butanoic acid | 10 | 0.317 ± 0.203 | 5 | 0.204 ± 0.048 |
|  | Pentanoic acid | 7 | 0.396 ± 0.487 | 4 | 0.300 ± 0.171 |
| Post-intervention (week 8) |  |  |  |  |  |
| All subjects | Acetic acid | 30 | 28.36 ± 7.54** | 29 | 25.11 ± 7.95** |
|  | Propanoic acid | 30 | 9.34 ± 4.13* | 28 | 6.67 ± 2.83* |
|  | 2-methyl-propanoic acid | 28 | 0.808 ± 0.379 | 26 | 0.804 ± 0.414 |
|  | Butanoic acid | 30 | 7.30 ± 4.42** | 28 | 6.94 ± 4.50** |
|  | 3-methyl-butanoic acid | 28 | 0.371 ± 0.191 | 24 | 0.348 ± 0.196 |
|  | Pentanoic acid | 22 | 0.743 ± 0.508 | 22 | 0.723 ± 0.407 |
|  |  |  |  |  |  |
| 6-18 mths | Acetic acid | 12 | 32.44 ± 5.98* | 10 | 25.48 ± 7.60* |
|  | Propanoic acid | 12 | 9.89 ± 4.98* | 9 | 5.80 ± 2.67* |
|  | 2-methyl-propanoic acid | 11 | 0.628 ± 0.406 | 7 | 0.677 ± 0.405 |
|  | Butanoic acid | 12 | 7.89 ± 5.13* | 9 | 4.23 ± 2.52* |
|  | 3-methyl-butanoic acid | 10 | 0.292 ± 0.202 | 5 | 0.250 ± 0.116 |
|  | Pentanoic acid | 7 | 0.529 ± 0.533 | 4 | 0.565 ± 0.566 |
| * *p* < 0.05 comparison between the placebo and LP N1115 groups.  ***p* < 0.05 comparison between the following time points (baseline vs. post- intervention).  n = the number of subjects in which SCFAs were detected. | | | | | |
